# Scene Classification for Sports Video Summarization Using Transfer Learning

**DOI:** 10.3390/s20061702

**Published:** 2020-03-18

**Authors:** Muhammad Rafiq, Ghazala Rafiq, Rockson Agyeman, Gyu Sang Choi, Seong-Il Jin

**Affiliations:** 1 Department of Information and Communication Engineering, Yeungnam University, Gyeongsan-si 38541, Korea; rafiq@ynu.ac.kr (M.R.); ghazala@ynu.ac.kr (G.R.); rocksyne@gmail.com (R.A.); 2 The division of computer convergence, Chungnam National University, Daejeon 34134, Korea

**Keywords:** deep learning, AlexNet CNN, small dataset, data augmentation

## Abstract

This paper proposes a novel method for sports video scene classification with the particular intention of video summarization. Creating and publishing a shorter version of the video is more interesting than a full version due to instant entertainment. Generating shorter summaries of the videos is a tedious task that requires significant labor hours and unnecessary machine occupation. Due to the growing demand for video summarization in marketing, advertising agencies, awareness videos, documentaries, and other interest groups, researchers are continuously proposing automation frameworks and novel schemes. Since the scene classification is a fundamental component of video summarization and video analysis, the quality of scene classification is particularly important. This article focuses on various practical implementation gaps over the existing techniques and presents a method to achieve high-quality of scene classification. We consider cricket as a case study and classify five scene categories, i.e., batting, bowling, boundary, crowd and close-up. We employ our model using pre-trained AlexNet Convolutional Neural Network (CNN) for scene classification. The proposed method employs new, fully connected layers in an encoder fashion. We employ data augmentation to achieve a high accuracy of 99.26% over a smaller dataset. We conduct a performance comparison against baseline approaches to prove the superiority of the method as well as state-of-the-art models. We evaluate our performance results on cricket videos and compare various deep-learning models, i.e., Inception V3, Visual Geometry Group (VGGNet16, VGGNet19), Residual Network (ResNet50), and AlexNet. Our experiments demonstrate that our method with AlexNet CNN produces better results than existing proposals.

## 1. Introduction

The rapid growth of devices and widespread internet connectivity and automation has generated data drastically like never before. In addition to textual data, video streaming contents are the main contributor in data generation, which demands a larger space and bandwidths to store and stream. The web pages with video content are more engaging and seem informative than that of only showing the text. Video processing, monitoring, summarizing, and broadcasting are snowballing with the introduction of live broadcast apps and the provision of live streaming on existing major video hosting websites. Therefore, the video processing is gaining the attention of the computer vision and deep-learning researchers. Video content searching, video description, and video summarization are the hot topics of the times. They are high-level tasks in the field of video processing. On the other hand, shot classification, scene classification, shot-boundary detection, and action recognition are the building blocks for those high-level tasks.

Sports videos are the most engaging and contributive content and have enormous commercial aspects. Sports video scenes are usually repetitive; because every sport has its predefined camera positions, zooming protocols, and action replay strategies. Sports videos are usually long in duration; therefore, it is not always possible for the offline audience to watch full contents of the recorded video for a specific event. Due to the longer duration of the recorded video and busier life of the audience, researchers are actively working on automatic video summarization, and scene classification is the core component for the sports video summarization. Since sports videos have known rules for video capturing and have a particular scene protocol, a sport-specific scene classifier can produce better results in comparison to generalized scene classifier. Sports video covers all the events, including exciting as well as boring events, and some of the events are less exciting, i.e., breaks, tedious parts of the game, and other activities where a viewer loses interest. It produces the opportunity to publish the videos consisting of only the best parts of the game. Therefore, accurate and high quality of scene classification can help to produce a high-quality video summary.

**Figure 1:**
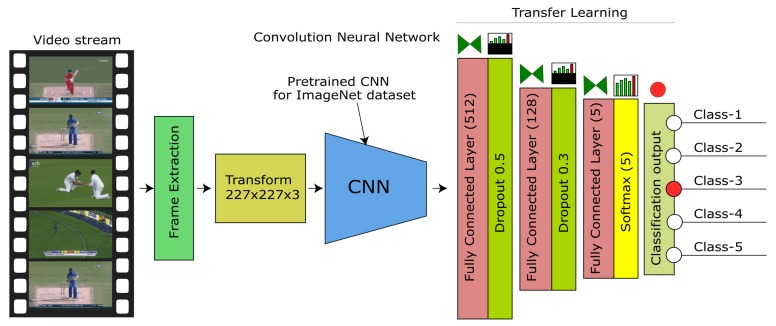
The proposed model architecture. Input video is extracted into frames and key frames are classified by a trained CNN. Output class identifies the scene and is compared if continuous video.

The objective of our proposed method is to present a high-performance method to achieve high-quality summarization. The lower error rate in classification ensures the high reliability of the classification and hence high reliability of the dependent processes that consume scene classification building block. We achieve this objective by introducing a three-step upgrade to the state-of-the-art method proposed by [1], i.e., proposed a particular transfer learning architecture, introducing the pre-trained weights for training the model and applying data augmentation for training data. The architecture of the proposed model for scene classification is shown in Figure 1. Our proposed model focuses on cricket as a case study, and we carry out our experiments on recorded videos taken from publicly available YouTube source.

The sports audience is more interested in meaningful content, and analyzing and summarizing the video content is a substantial labor task. Computers are helping humans more promising than ever before, and particularly with the evolution of machine learning and other methods of artificial intelligence (AI) in almost every domain of the life sciences. Therefore, researchers are proposing novel techniques almost all the time. Since scene classification if a fundamental building block, it requires significant attention to improve the performance over the existing research state-of-the-art level. Previously, several researchers proposed generalized schemes for shot classification, shot-boundary detection, and scene classification; however, generalized proposals are less helpful for a specific domain. Proposing a specialized technique has its significance and achieves superiority over the existing generalized proposals.

Many researchers proposed excellent methods recently with significant improvements. Minhas et al. [1] proposed a method based on deep learning techniques and presented a bunch of improvements. However, a quality gap is still present, and a requirement exists to fulfill this gap in addition to practical implementation limitations. Due to the non-availability of labeled sports datasets in this domain and context, improvement is necessary to produce high-quality classification on a smaller dataset in practically less time.

**Figure 2:**
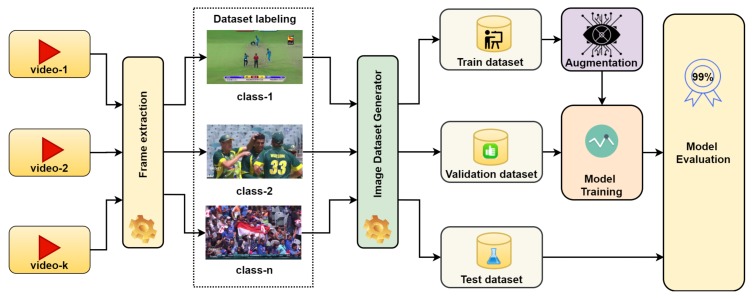
Proposed model training and evaluation block diagram.

In this paper, we propose a model for sports video scene classification with the particular intention of sports video summarization. The proposed method contributes a combination of three techniques over the existing state-of-the-art technique [1]: the proposed architecture for the classification model, incorporating the pre-trained weights and training data augmentation. In the proposed architecture, we present a set of new layers for the transfer learning of AlexNet CNN. The proposed architecture is designed to support five critical scene classes required in the sport of cricket. We introduced additional fully connected layers with dropout and activation functions. We prepare the cricket sports dataset from YouTube cricket sports recorded videos from the past events. We label the five most frequent and essential scenes from the videos, i.e., batting, bowling, boundary, close-up, and the crowd, to evaluate it to be the best fit for video summarization of cricket sport. Figure 2 shows the training procedure. We load the model with pre-trained weights on ImageNet challenge with 1000 classes and then confined the feature map to our five classes by using the additional proposed layers. We split the training image data into the thee subsets for training, validation, and test. We augment the training data using geometric transformations. We employ the in-place data augmentation technique and introduce a large image repository using batches of 32 images at a time. In-place transformations do not increase the size of the training data; hence control over training time is achieved, whereas increasing training dataset size potentially increases the training time for the model. We iterate several learning rates to find the best learning rate to achieve peak accuracy. We perform our experiments using the best learning for the latest deep-learning models on the same dataset.

We compare the model with the existing state-of-the-art models, particularly deep-learning models, e.g., Inception V3, VGGNET16, VGGNET19, ResNet50, and from non-deep-learning models like SVM to present a performance evaluation. We also compare the model with existing models in the field of sports video summarization and scene classification. We focus on scene classification with a primary intention of video summarization using deep-learning methods, and we have selected the sport of cricket as a case study.

Our proposed methodology demonstrates a 5% better performance over the existing method proposed by [1] and supersedes the accuracy, precision, and recall with a significant margin of 4–5% in all parameters. We achieve a mean accuracy of 99.26% with a stable precision of 99.27% and a reliable recall of 99.26%, which yields the F1-measure of 99.26% to show the high quality of the classification. Furthermore, since we incorporate pre-trained CNN in the proposed method, consequently, it reduces the training time of the model drastically. On the other hand, pre-processing of the training data by introducing augmentation, eliminates the overfitting, and helps to achieve the high quality of the model state. We introduce three fully connected layers appended to pre-trained CNN and applied the best learning rate during our extensive experiments. Our methodology shows superior performance over existing methods during our experiments.

The primary audience of the proposed method is the video summarization and video description researcher community. However, classification, in general, can also be employed in several other domains, i.e., medical image analysis [2,3], agriculture classification [4], biological organism classification, self-driving vehicles [5], video captioning and other similar domains.

The rest of the paper is organized into the following four sections, Section 2 covers the related work in the subject domain. The proposed method is presented in Section 3. The experimental results and discussion is detailed in the Section 4 and finally the Section 5 conclude the work and suggest the future aspects.

## 2. Related Work

Existing approaches presented significant work on sports videos. Mainly shot classification and scene classification methods classify the camera views in the field, i.e., audience, players, and state of the sports field views specialized to each sport. Recent approaches employed various methods and models, including conventional classification models, i.e., Kth Nearest Neighbor (KNN), Support Vector Machines (SVM), as well as deep-learning models, i.e., AlexNet CNN, Recurrent Neural Network (RNN), Long Short-Term Memory (LSTM), Visual Geometry Group Network (VGGNET) and Gated Recurrent Units (GRUs). Approaches involving deep structures ensure an improved accuracy but at the cost of increased computational complexity. The need for a dedicated dataset for large-scale multi-label video classification is being addressed by Abu-El-Haija et al. [6], with over eight million videos summing to 500,000 h of playtime and 4800 visual attributes. The videos were labeled using vide annotation system from YouTube™. They classified videos into vehicles, sports, and concerts. We will review both classical and deep-learning approaches applied for scene classification in sports videos.

### 2.1. Scene Classification via Conventional Approach

Rahul et al. [7] presented a soccer video frame classification using multi-class kernel SVM. A window-based shot-detection scheme is adapted. For a decided window size, the difference of a specific frame is calculated with every other frame. Depending upon a particular threshold, that specific frame is declared to be a shot-boundary. The shot can be classified as one of Bowler Runup, Batsman Stroke, Player Close-Up, Umpire, Ground, Crowd, Animations, and Miscellaneous. Temporal neighbor-hood information is also catered while performing the classification. Dataset used is collected from YouTube regarding the Indian Premier League (IPL) tournament consisting of 16 matches with a total of 64 h of playtime. Zhou L. [8] proposed a moving sports video moving object detection and tracking using the hidden Markov model (HMM) to track the players in the ground using a top-view camera for the tracking of moving players. The integration of the hidden Markov model in physical education can reform physical education for students. The applied method maintains incredible accuracy and stability throughout the tracking process. Ling-Yu et al. [9] proposed a unified framework for semantic scene classification in sports videos. The proposed method implements supervised learning for top-down video scene classification. Low-level features were derived using color, texture, and DC images in the compressed domain. These low-level features were converted to mid-level features such as camera motion, action regions, and field shape properties. Mid-level representation was necessary to use the classifiers that do not map on low-level features. Finally, shots were classified using different supervised learning algorithms like decision trees, neural networks, support vectors machine (SVM). Camera shots were classified into mid shots, long shots, and close-up shots. The proposed framework achieved a classification accuracy of 85% to 95% on five different sports, i.e., volleyball, basketball, tennis, soccer, and table tennis. Ashok et al. [10], proposed an optical flow approach for different sorts of batsman stroke detection in cricket sports videos. A histogram-based technique is being adopted to detect the shot-boundary. Low-level features (i.e., grass pixel ratio) and mid-level features (i.e., camera view, distance, etc.) were used to train a hybrid classifier to classify seven classes, i.e., ground view, batsman view, crowd view, long shot, pitch view, fielder view, and others. The proposed approach comprises Naïve bias, KNN, and multi-class SVM for shot classification into field-view and non-field views. The method used 29,000 frames from cricket match video played between England and Australia for training and 14,000 frames for testing purposes. The authors claimed for 90.9% of recall with 50% precision. Overall accuracy following the optical flow mechanism is around 80%. Sigari et al. [11] exploited the heuristic/ rule-based technique as well as employed SVM for counter-attack detection based on camera motion estimation. The first step in the process of counter-attack detection detects the shot boundaries and makes segments of the video to the detected shots. An SVM-based classifier with RBF kernel produced the best results.

### 2.2. Scene Classification via Deep-Learning Approach

Minhas et al. [1] proposed a deep-learning-based shot classification model and presented good accuracy over different shot classes consisting of long, medium, close-ups, and crowd/out-of-field shots. A well-known deep-learning model, AlexNet CNN is used to classify shots or frames extracted from the sports videos and employed four classes to train the network from scratch on Nvidia GTX 580 Graphic Processing Units (GPU). Overall training and validation performance are boosted using response normalization and dropout layers. The adopted methodology demonstrated good accuracy of 94.07% against baseline methods on the subjected dataset. Russo et al. [12] proposed a model by combining deep learning and transfer learning, i.e., combining the functionality of VGGNET16, RNN, and GRU with transfer learning for final classification. The authors hand-drafted the sports dataset to focus on sports action-based classification. These mixed sports videos were used to classify 10 and 15 target classes using CNN as feature extractor and then combining with temporal information from RNN for model formulation. The model demonstrated a good accuracy of 94% for 10 and 92% for 15 sports classes. Ng et al. [13] is capable of handling full-length videos using the AlexNet and GoogLeNet models. Sports-IM consisting of around 1.2M YouTube sports videos with 487 classes and UCF-101 containing 13,320 videos with 101 action classes are used while evaluating the proposed architecture. Video frames are augmented for diverse training of the model. For LSTMs, the optical flow mechanism showed improvement in the results, whereas the fusion of convolutional pooling networks with optical flow did not show any improvement. Jungheon et al. [14] proposed a multi-Modality classification system for video events using visual and audio relationships extracted from the video. Inception-V3 algorithms are employed for image feature vector extraction. However, Mel Frequency Cepstral Coefficients (MFCCs) were used to extract the audio feature vector. Finally, SVM and SoftMax classify video events. YLI-MED dataset with 1823 videos and a self-collected YouTube dataset comprising of 1369 videos were used for evaluation of the proposed system. Normalization of audio and visual feature vectors, as well as correlation integration, achieved better performance results. Hong et al. [15] proposed an end to end Soccer video scene and event classification using CNN with Deep transfer learning and achieved above 89% of accuracy. Separable features extracted using the CNN model can be visualized in 3D space. Transfer learning was applied to avoid overfitting of the model. A new Soccer Video Scene and Event dataset (SVSED) is introduced, comprising of 600 video clips classifying six classes. Agyeman et al. [16] proposed sports video summarization by considering spatiotemporal features learning using 3D CNN and LSTM by employing fine-tuning. CNN based on Residual Network (ResNet) is used for feature extraction from video clips. Summarization of 10 soccer videos evaluated by 48 participants from 8 countries was performed. The self-annotated dataset, as well as UCF101 dataset comprising of 13,320 video clips with 101 human action classes, is used to evaluate the performance of the system on five classes, i.e., centerline, corner-kick, free-kick, goal, and throw-in. Video summarized using the proposed system received a good score of 4 out of 5 from the participants. Sozykin K et al. [17] proposed a 3D CNN-based multi-label deep Human Action Recognition (HAR) system for sports video summarization for the sport of Hockey and presented more than ten classes. Data pre-processing techniques like resizing, normalization, windowing, and sequence labeling were used. The dataset used for evaluation contains 36 grayscale videos recorded with a static camera position. The authors obtained a low mean F1-score of 67% on the single multi-label k-output model (SMKO). Bhalla et al. [18] proposed a multimodal approach for automatic cricket video summarization. They employed Optical Character Recognition (OCR), sound detection, and replay detection techniques for the extraction of essential events in the cricket match. The proposed system achieved an accuracy of 89.45% for event detection. Highlights generated by the system were compared with the highlights telecasted by the official broadcaster of the match. Tejero-De-Prablos [19] presented a scheme for user-generated sports video summarization and employed the LSTM model to identify various user actions in the video. Videos were classified into interesting or non-interesting categories. The performance of the system compared with several combinations of different features. Evaluation results outperform existing summarization techniques. Shih [20] covered various content-aware sports video analysis and summarization techniques on a broader scope of games, techniques, issues, benchmarks, and datasets. Karmaker et al. [21] employed an optical flow tracing method for shot classification of the cricket sport. A trained 3D MACH filter recognized the actions. A filter trained over six videos to recognize the actions was employed. Finally, Decision trees and ELMAN neural network was used to classify the shots.

## 3. Proposed Method

This section provides a detailed methodology of the proposed method. The core of the proposed method is AlexNet CNN, which is pre-trained on ImageNet [22] weights. Figure 1 shows the architecture of the proposed model. We employed AlexNet CNN as a foundation with pre-trained weights and employed transfer learning by adding three fully connected layers and trained our model on the labeled sports dataset. The proposed method consists of five steps. In step 1 we prepared the dataset from publicly available sports videos, then in step 2, we employed AlexNet CNN and added three additional fully connected layers. First two layers appended with dropout and last layer activated with SoftMax, as shown in Figure 1, and after the layer configuration, we compiled the model with extended layers. In step 3, we loaded pre-trained weights to the model. In step 4, we split the dataset for training, validation, and testing, and applied augmentation on training data. Finally, in step 5, we trained the model. Figure 2 shows the block diagram of the training and evaluation procedure.

### 3.1. Proposed Model Architecture

In the proposed method, we employed AlexNet CNN deep-learning architecture for scene classification and evaluated using Keras application 2.0 and Tensor Flow backend in Python. Keras provides a variety of deep-learning application layers. Table 1 enlists the complete layer configuration of the proposed model.

#### 3.1.1. Application Layers

We employed Keras layers to construct AlexNet and extended the codebase from the ConvNet library [23]. AlexNet CNN then loaded pre-trained weights from [24]. The proposed layer architecture consists of Keras ConvNet AlexNet model from layers 1 to 32 and the transfer learning from layers 33 to 38. Layer 37 consists of five neurons to match our classification task. The dense layer with five output classes confines the probabilities received from the dropout-5 layer, and then output is activated with SoftMax activation. Final classification based on probabilities obtained from SoftMax activation is done to find the exact association from the output heat-map. The AlexNet CNN model requires input as a 3D tensor with a size 227×227×3 for color images to be introduced to the model for training and classification.

#### 3.1.2. Convolutional Layers

Convolution is the primary process of any neural network model required for learning and classification using feature maps. In this layer, a kernel (a small matrix with specialized values to extract features) is convoluted over layer input tensor to obtain feature maps and forwarded to output tensor. We obtain an output feature map *T* from the Equation (1) and (2).

(1) $$T_{h}^{r}=\frac{T_{h}^{r-1}-K_{h}^{r}+2P}{S_{h}^{r}}+1$$

(2) $$T_{w}^{r}=\frac{T_{w}^{r-1}-K_{w}^{r}+2P}{S_{w}^{r}}+1$$

Where *T* is the output tensor with $T_{w}$ width and $T_{h}$ height of the feature map of the previous layer. *K* denotes the kernel with size $K_{w}$ and $K_{h}$ and filter slides with stride *S* to $S_{w}$ and $S_{h}$ horizontal and vertical directions with padding *P*. Similarly, *r* indicates the layer of operation. Output feature map tensor is obtained by applying a convolution process on the input layer *X* by kernel *K* such that Y(x,y)=(X∗K)(x,y) where Y(x,y) is the output feature map tensor and product of the input tensor *X* and the kernel *K* with *x* and *y* spatial indices. The convolution is represented here with standard notation by symbol ∗. Equation (3) shows the detail expression of convolution in Equation (3)

(3) $$Y\left(x,y\right)=\sum_{i=-\frac{Kw}{2}}^{\frac{Kw}{2}}\sum_{j=-\frac{Kh}{2}}^{\frac{Kh}{2}}X\left(x-i,y-j\right)K\left(i,j\right)$$

The proposed framework consists of five convolutional layers with three split convolutional layers to load balance on multiple GPUs. We have eliminated batch normalization after convolutional layers since we have not observed any accuracy improvements during our experiments. Convolutional Layers’ output was activated using Rectified Linear Unit (ReLU)

#### 3.1.3. Transfer Learning Layers

A fully connected layer cross-connects every neuron from the input tensor to every neuron in the output tensor, with an activation function at the output layer. The fully connected layer flattens the input tensor if the input shape rank is higher than 2. Hence a fully connected layer is a dot product of the input tensor by the applied kernel.

Fully connected layers generate probabilities in a wide range; therefore, to reduce the noise level, it is essential to eliminate the weaker predictions. To perform this operation, a dropout process is carried out with a certain threshold of usually 0.5. This less densifies the neurons after removing the value of lesser probabilities. The dropout layer helps to avoid a model overfitting and significantly improves the validation accuracy. A dropout layer reduces the functional size of the process by removing unproductive neuron outputs. It is a regularization technique and filters out complex co-adaptation during the training phase.

#### 3.1.4. Activation Function

Rectified Linear Unit (ReLU) is a tensor output activation function and makes the model training process non-linear. During the convolution process output, tensor may contain positive and negative values; therefore, before forwarding the output to the next layer, an activation function is applied. A ReLU function converts the values lower than zero to a zero value, and positive values leave unchanged. This process is called rectification. A non-saturating function f(x)=max(0,x) returns zero or positive values from a range of negative and positive values. The output feature map is cleaned out of negative values. It increases the non-linearity of the model without compromising the quality of classification in receptive fields during the convolution process. We can express ReLU function mathematically, as shown in Equation (4).

(4) $$Y\left(x,y\right)=\left\{\begin{array}{cc} 0, & X(x,y)\leq 0\\ X(x,y), & otherwise \end{array}\right.$$

Where X(x,y) is the input of ReLU, and Y(x,y) is computed output from ReLU activation for a specific neuron at *x* and *y* position.

### 3.2. Data Augmentation

In the proposed method, data augmentation plays a significant role in improving the performance of the model. Data augmentation relates to modifying the image data and perform a series of operations, such that the modified image remains in the same class. Data augmentation helps to generalize the input data, which, as a result, reflects a better test accuracy. There are three main techniques while augmenting training data: expanding dataset, in-place or on the fly augmentation, combining dataset with in-place augmentation. In our experiments, we employed in-place data augmentation.

In-place augmentation relates to modifying the original data by applying a series of transforms and returns the transformed data only. It exceptionally means that the size of the data is not changed, and source data is not permanently modified, hence, it is not an additive process. Therefore, in repeating experiments, the training dataset is always different from the previous training since the data is transformed in-memory batch by batch with random transformations before inputting for the training of the model.

We employed Keras ImageDataGenerator to augment our training data. It provides various transforms to augment the image data such that scale, rotate, shear, brightness, zoom, channel shift, width and height shift, and horizontal and vertical shift. In the proposed method, we applied geometric transforms like rescale, rotation, width shift, height shift, and horizontal shift to augment our source data for our experiments. In our experimental setup, on each input batch, we applied the rotation angle of 30 degrees, width, and height shift each 20% and horizontal flip randomly.

The augmentation block in fig-training accepts training source data from the data generator as a batch. It applies a series of random transformations as described above on each image in the batch. The transformed image batch replaces the original image batch and is presented to input for the training generator.

### 3.3. Implementation Details

In the proposed model, we introduced additional fully connected layers for the fine-tuning of the model. Configuration and implementation of the complete AlexNet CNN as a foundation model helped to load pre-trained weights, and appending additional layers does not disturb the carefully trained ImageNet weights. We employed three fully connected layers, i.e., dense-4 and dense-5 layers followed by dropout of 50% and 30% respectively, and the last, i.e., dense-6 layer, followed by a SoftMax activation layer. The fully connected layers were employed in an encoder fashion, with layer shapes 512, 128, and five, respectively. Training time was observed to be quite low with respect to the time for training the network from scratch. Table 2 shows the transfer learning layers configuration. We employed additional layers to tackle more classes with inter-class similarities and intra-class differences.

Deep learning has rapidly taken over the complex classification tasks for intelligent applications [25]. In the image classification domain [26], it requires enormous time to train a network up-to its optimal weights; however, transfer learning [27] has shown promising improvements in training time and contextual classification. Transfer learning can be adapted in three ways. 1: Fixed feature extractor, in which last fully connected layer is replaced to classify frames based on trained network, 2: Fine-Tuning of the CNN relates to adjusting the weights to fit the model in the custom scenario; this is done using backpropagation, which can be applied on almost any layer of the AlexNet CNN, 3: Partial Pre-trained models are shared by the community. Checkpoints during the model training are obtained, which are shared with other researchers to save time. Transfer learning reduces the training time, in most of the cases. In the proposed scenario, all the layers of AlexNet pre-trained network remained unchanged and employed the complete model. We employed the model adapted from AlexNet, loaded the pre-trained weights, and appended new fully connected layers.

Training a model from scratch requires a large dataset to obtain reasonable accuracy, which, however, requires a significant time and processing cost, whereas using a small dataset causes the model overfitting. Introducing data augmentation strategy boosts the model validation accuracy by approximately 10% [28], and combining data augmentation with pre-trained weights, trains a model near-perfect even if the dataset is not very large. It reduces the training time and achieves higher evaluation scores.

Video Source is an MPEG-4 digital video of the full-length recorded match. It contains all the features that a live stream represents. A video is a composition or group of frames, and a frame is an image with the same size as the source video display size. The frame extraction process takes one frame at a time for further processing. We extracted frames at six frames per second from the video, such that we picked every fifth frame from a video with a frame rate of 30 frames per second. A large number of frames are obtained from a video of a full event. The frames obtained from the video are high resolution and landscape. So before feeding a frame to the classification system, frames were resized to match the input shape of the model, i.e., 227×227×3. The pre-conditioned frames with RGB color space were fed into the input layer of the classifier model.

Pre-conditioned frames are introduced to input in the proposed CNN model, which classifies the input frame to a target class. Labeling the classes correctly for event detection is particularly important since the classification results highly depend on the model training accuracy. Scene classification is based on frame classification and is performed over a range of frames detected for a certain predefined class. A temporal similarity between consecutive frames results in the same class until there is a significant change in the scene.

## 4. Results and Discussion

The result section represents the experimental evaluations of the performance of the proposed methodology. In the later part, we will discuss the results in detail. We evaluated our results using precision, recall, accuracy, error rate, and F1-score for all available categories on the dataset of videos. We employed various small and large datasets sourced from publicly available datasets for image classification and manually labeled dataset of sports videos. However, we have presented results for the sports video dataset only.

### 4.1. Dataset

For performance evaluations, we have selected various sports videos from YouTube™, considering different series and different lighting conditions. Our dataset consists of the following videos. Cricket 2014 New Zealand v South Africa 1st ODI Highlights - YouTube [29], World Record 438 Match-South Africa vs. Australia- Part 2 - YouTube [30], Pakistan vs. Australia 3rd ODI Full Highlights - YouTube [31], 1st ODI: Pakistan vs. Australia HD Full Highlights - YouTube [32].

**Figure 3:**
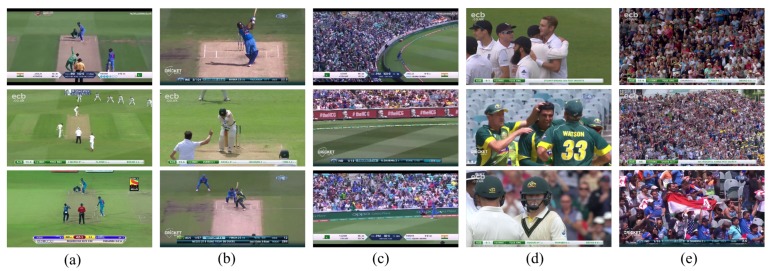
Scene classes, (**a**) Bowling, (**b**) Batting, (**c**) Boundary, (**d**) Close-up, and (**e**) Crowd.

A collection of 6800 frames was extracted from the source videos and labeled for five required classes. Figure 3 depicts the various classes employed in our experiments. The proposed method can classify different sports; however, to validate the method performance, we considered cricket as a case study. Frame groups from key-events of the cricket sport were chosen as classes to identify the shots and scenes expertly. The extracted frames were purposely classified to support video summarization tasks for the sport of cricket. Due to the nature of video data, there are several features where overlaps are present in multiple classes, e.g., almost all the classes have people (audience, empire, and players) in view; furthermore, bowling and batting have the pitch as a standard feature. Boundary and crowd have the same size of the audience in both classes, and batting and close-up classes also portray inter-class similarities. On the other hand, due to the various colors of shirts in a single scene, there are intra-class differences, which makes the dataset a problematic task for classifiers, especially when there is a smaller dataset. Our proposed framework handles it effectively using augmentation and taking advantage of pre-trained weights to achieve robust performance over a shorter time for training in addition to a smaller dataset.

### 4.2. Training Procedure

**Figure 4:**
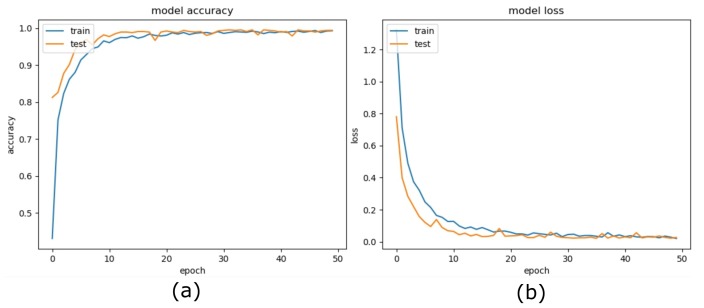
Proposed model training history. (**a**) Model accuracy, (**b**) Model loss.

**Figure 5:**
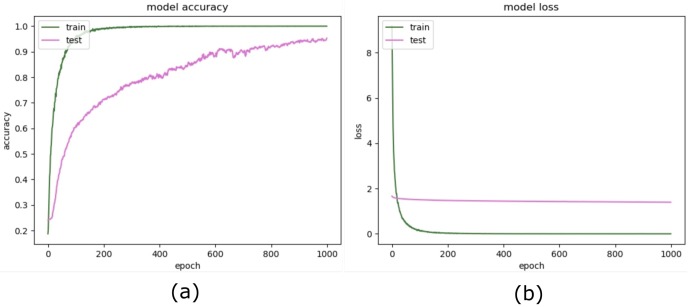
Minhas et al. training history. (**a**) Model accuracy, (**b**) Model loss.

Figure 2 explains in detail, the complete process of the model training and evaluation of the proposed method. We employed TensorFlow™ backend with Keras applications for training purposes and incorporated dataset generators to handle the large dataset of images with optimal memory usage. Image data was fed to the training classifier using a batch of 32 images augmented with the Keras ImageDataGenerator class [33]. We parameterized the generator to transform input images to apply a set of transformations such that flip, rotate, resize, and shift at both dimensions. We partitioned dataset into 70%, 30% for training and testing respectively, and separated 15% from training for validation dataset. Training achieved a robust accuracy of above 99% in very initial epoch; however, to observe the overfitting and stability, we executed training until 50 epochs. We applied the Adam optimizer, a learning rate of 0.00001, and a loss model of ’categorical cross-entropy’ with accuracy as the training performance metric. Figure 4 shows the training progress over 50 epochs and reflects the stability and high achievements of our proposed model, whereas Figure 5 depicts the training history of the model proposed by Minhas et al. [1].

### 4.3. Simulation Parameters

During our simulations, we applied optimistic and realistic parameters, considering the video summarization. We selected Python as a primary programming language throughout the simulation process. We uploaded pre-trained weights from ImageNet with 1000 classes and used Adam optimizer with an optimal learning rate for training optimization. Table 3 lists the simulation parameters used in our experiments.

### 4.4. Learning Rate Selection

**Figure 6:**
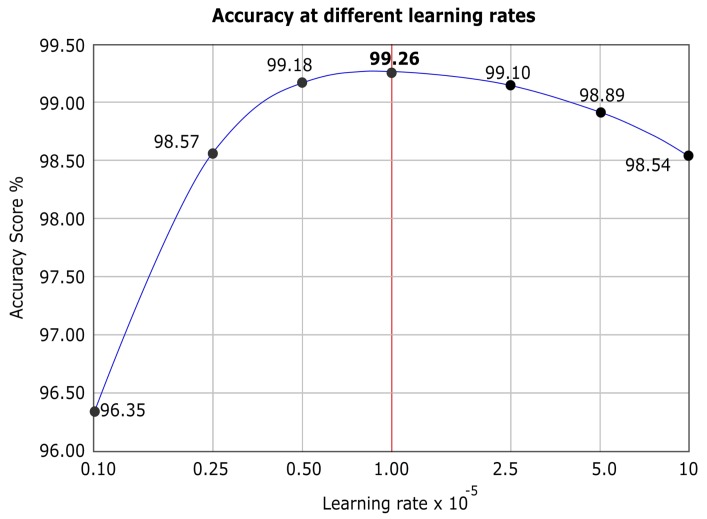
Accuracy obtained using different learning rates.

During the experiments, to train and evaluate the model, various learning rates were tried. The best learning rate under Adam optimizer worked with 0.00001. To keep the parameters comparable, we applied the same learning rate and the same optimizer for all the models under evaluation. Figure 6 depicts a logarithmic line graph to show the accuracy response obtained from the model at different learning rates.

### 4.5. Evaluation Metrics

We evaluated our experiments using standard model evaluating metrics. We measure correct decisions and erroneous predictions in terms of precision, recall, and F1-score. F1-Score is the harmonic mean of precision and recall. It reaches its best value at 1 when precision and recall both are perfect, whereas the worst F1 score is zero. It gives a better measure of incorrectly classified cases. Equation (5) expresses the F1-score as a measure of model performance.

(5) $$F1Score=2*\frac{\left(Precision*Recall\right)}{\left(Precision+Recall\right)}$$

### 4.6. Performance Evaluation

**Figure 7:**
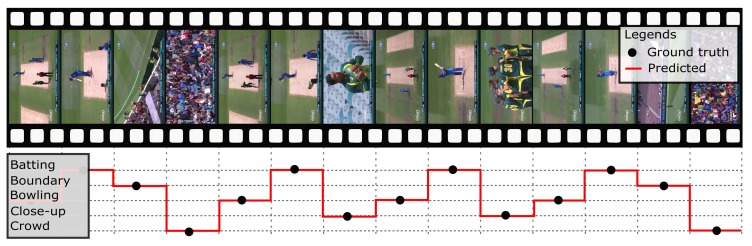
Video scene classification predicted vs. ground truth.

The proposed model presented improvements in accuracy, precision, recall, and F1-score. The model achieved a robust performance in comparison to other models. We compared our model with other deep CNN models like Inception v3, ResNet50, VGGNET16, VGGNET19 as well as Support Vector Machine (SVM) model and with the work presented by Minhas et al. The performance demonstrates the robustness of the model. The proposed model achieved a mean accuracy of 99.26%, precision of 99.27%, recall of 99.26%, which computes to 99.26% of F1-score, and an error rate of 0.74%. Results indicated the effectiveness of the proposed model in the domain of sports video summarization. Figure 7 demonstrates the predicted classification of the scenes compared with ground truth.

Proposed model evaluations reveal the inter-class similarities and intra-class differences. Table 4 presents the confusion matrix of incorporated classes for shot classification. Inter-class similarities are evident in bowling and batting; however, even though there are significant similarities between boundaries and crowd due to the audience shown in the long-distance camera view, the proposed model classified it precisely. However, Table 5 reflects the confusion matrix presented by [1] and is a comparatively an under-optimized matrix even after 1000 epochs in contrast to 50 epochs for our proposed model. We evaluated various models shown in Table 6 to compare model performance against each class. Performance indicator F1-score shows that the proposed model outperformed among other deep CNN models, as well as SVM.

### 4.7. Performance Comparison with Existing Sports Video Shot/Scene Classification Methods

We compared the performance of the proposed model with state-of-the-art models and existing methods proposed in recent research in sports video shot classification. Table 7 shows the results summary and quality score.

To compare our model, we selected the most relevant and recent work in the same domain. Minhas et al. [1] proposed a deep CNN model with significant performance improvement against state-of-the-art methods like SVM, KNN, and ELM. They employed cricket and soccer mixed images for a dataset of four classes (close, medium, long, crowd). They prepared dataset from YouTube videos by frame extraction, labeled it manually and trained the network from scratch. The last fully connected layer was replaced with the required number of categories and trained the network over a set of categories from a collection of frames extracted from the publicly available video sources. The authors introduced frames of multiple sports like baseball, soccer, and cricket. It improved the overall classification capability of CNN Spectacularly lower inter-class similarities. To fairly compare our proposed model with the reference model [1], we have implemented the reference model to test the performance of both methods using the same dataset. Table 7 shows performance comparison and superiority of the proposed model is evident.

Various studies proposed models, frameworks, and methodologies in the recent past for shot classification exclusively or part of their scheme, conclusively, deep-learning approaches presented high quality of the classification. Deep learning proved its importance in image classification, and incorporating the pre-trained model and applying augmentation [28] improved results drastically and reduced training time. Our experiments over sports videos scene classification using the above techniques in addition to appending three fully connected layers in a pyramid shape to converge output to five classes exhibited the improved classification results. This combination of multiple techniques together made this model superior in terms of training time and the size of the dataset required for training and the performance. We employed a high-power GPU for our simulations, which helped us run various simulations to find the best learning rates, evaluate several deep-learning models, and process video data efficiently.

We selected the best learning rate to achieve the peak performance of the model. We tested our model on various datasets publicly available on the Kaggle [34] to make sure it maintains superiority over reference-models. We performed our experiments on a handpicked dataset extracted from the video streams. The results are improved over the existing techniques. However, there are certain limitations to the method, i.e., the frames that do not belong to any category get easily miss-classified to the closest target class. Such frames are not necessary for summarization, especially if they were ignored during the training process by the authors, but such frames cause a noise. To use this technique in video summarization, we suggest training the model by including all possible available scene classes. We suggest using this method in conjunction with shot-boundary detection methods to produce excellent video summarization performance.

## 5. Conclusions and Future Work

In this paper, we proposed a model for sports video scene classification with the particular intention of sports video summarization. Our findings demonstrated that our proposed model performed best against state-of-the-art models and existing recent research level. We evaluated our proposed model on cricket as a case study and obtained videos from publicly available YouTube source. We labeled the five most frequent scenes from the videos, i.e., batting, bowling, boundary, close-up, and the crowd to evaluate it to be the best fit for video summarization. We compared our proposed model with the existing state-of-the-art models, particularly deep-learning models, e.g., Inception V3, VGGNET16, VGGNET19, ResNet50, and from non-deep-learning models like SVM to present a performance evaluation. We also compared our proposed model with recently proposed models in the field of sports video summarization, and scene classification; the performance results showed a remarkable improvement.

Although the proposed methodology is evaluated over sports video data to provide a building block for video summarization; however, it can also be used for medical image analysis, agriculture classification, biological organism classification, self-driving vehicles and various other fields where classification requires respectively higher accuracy and the error rate is a critical constraint. The proposed model produced excellent performance indicators over previously proposed models, which makes it a strong candidate to be a reusable building block. Scene classification can be further extended to research on video description and medical video analysis and event description. Similarly, sports video to text summarization is a hot topic of the times and requires researchers to improve the quality of the task.

## Figures and Tables

**Table 1:** Proposed model architecture (Transfer Learning).

S#	Layer (Type)	Output	Parameters
1.	input_1 (InputLayer)	(None, 3, 227, 227)	-
2.	conv_1 (Conv2D)	(None, 96, 55, 55)	34,944
3.	max_pooling2d_1 (MaxPooling2D)	(None, 96, 27, 27)	-
4.	zero_padding2d_1 (ZeroPadding2D)	(None, 96, 31, 31)	-
5.	lambda_1 (Lambda)	(None, 48, 31, 31)	-
6.	lambda_2 (Lambda)	(None, 48, 31, 31)	-
7.	conv_2_1 (Conv2D)	(None, 128, 27, 27)	153,728
8.	conv_2_2 (Conv2D)	(None, 128, 27, 27)	153,728
9.	conv_2 (Concatenate)	(None, 256, 27, 27)	-
10.	max_pooling2d_2 (MaxPooling2D)	(None, 256, 13, 13)	-
11.	zero_padding2d_2 (ZeroPadding2D)	(None, 256, 15, 15)	-
12.	conv_3 (Conv2D)	(None, 384, 13, 13)	885,120
13.	zero_padding2d_3 (ZeroPadding2D)	(None, 384, 15, 15)	-
14.	lambda_3 (Lambda)	(None, 192, 15, 15)	-
15.	lambda_4 (Lambda)	(None, 192, 15, 15)	-
16.	conv_4_1 (Conv2D)	(None, 192, 13, 13)	331,968
17.	conv_4_2 (Conv2D)	(None, 192, 13, 13)	331,968
18.	conv_4 (Concatenate)	(None, 384, 13, 13)	-
19.	zero_padding2d_4 (ZeroPadding2D)	(None, 384, 15, 15)	-
20.	lambda_5 (Lambda)	(None, 192, 15, 15)	-
21.	lambda_6 (Lambda)	(None, 192, 15, 15)	-
22.	conv_5_1 (Conv2D)	(None, 128, 13, 13)	221,312
23.	conv_5_2 (Conv2D)	(None, 128, 13, 13)	221,312
24.	conv_5 (Concatenate)	(None, 256, 13, 13)	-
25.	convpool_5 (MaxPooling2D)	(None, 256, 6, 6)	-
26.	flatten (Flatten)	(None, 9216)	-
27.	dense_1 (Dense)	(None, 4096)	37,752,832
28.	dropout_1 (Dropout)	(None, 4096)	-
29.	dense_2 (Dense)	(None, 4096)	16,781,312
30.	dropout_2 (Dropout)	(None, 4096)	-
31.	dense_3 (Dense)	(None, 1000)	4,097,000
32.	dropout_3 (Dropout)	(None, 1000)	-
33.	dense_4 (Dense)	(None, 512)	512,512
34.	dropout_4 (Dropout)	(None, 512)	-
35.	dense_5 (Dense)	(None, 128)	65,664
36.	dropout_5 (Dropout)	(None, 128)	-
37.	dense_6 (Dense)	(None, 5)	645
38.	softmax (Activation)	(None, 5)	-

Model layer architecture shows the layers as produced from the Python program. The layers from 1 to 32 represent the AlexNet CNN base model and is compatible with ImageNet weights available at [24]. The layers from 33 to 38 are proposed transfer learning layers. Fully connected layers (33, 35) are activated with Rectified Linear Unit (ReLU) function and last layer at 37 is activated with SoftMax for classification output.

**Table 2:** Transfer Learning - Layer configuration.

S#	Layer (Type)	Output	Parameters
33.	dense_4 (Dense)	(None, 512)	512,512
34.	dropout_4 (Dropout)	(None, 512)	-
35.	dense_5 (Dense)	(None, 128)	65,664
36.	dropout_5 (Dropout)	(None, 128)	-
37.	dense_6 (Dense)	(None, 5)	645
38.	softmax (Activation)	(None, 5)	-

**Table 3:** Simulation parameters.

Model	Proposed Method, ResNet50, Inception v3, VGG16, VGG19	Minhas et al.	SVM
Language	Python, Keras	Python, Keras	Python, Keras
Pre-trained	ImageNet, 1000 classes	No	No
Optimizer	Adam Optimizer	Adam Optimizer	RBF Kernel
Learning rate	0.00001	0.0000001	gamma [0.001, 0.0001]
Loss function	Categorical Cross-Entropy	Categorical Cross-Entropy	
Performance metric	Accuracy	Accuracy	Accuracy
Total classes	05	05	05
Video file format	MPEG-4	MPEG-4	MPEG-4
Augmentation	Scale, Rotate, Shift, Flip	None	None
Batch size	32	32	NA

**Table 4:** Confusion matrix (proposed model).

	Predicted				
**Actual**	**Batting**	**Boundary**	**Bowling**	**Close-Ups**	**Crowd**
Batting	184	0	4	0	0
Boundary	0	459	0	0	0
Bowling	10	0	351	0	0
Close-ups	0	0	0	791	0
Crowd	0	0	0	0	90

The confusion matrix for the proposed model shows a high-quality classification over five classes.

**Table 5:** Confusion matrix Minhas et al. [1].

	Predicted				
**Actual**	**Batting**	**Boundary**	**Bowling**	**Close-Ups**	**Crowd**
Batting	169	4	2	13	0
Boundary	0	451	0	8	0
Bowling	25	3	300	33	0
Close-ups	1	3	0	787	0
Crowd	0	17	0	2	71

The confusion matrix shows a high rate of frame misclassification.

**Table 6:** Classification F1-score comparison with state-of-the-art models.

Model Names	Epoch	Batting	Boundary	Bowling	Close-Ups	Crowd	Mean
Minhas et al.	1000	88%	96%	90%	96%	88%	92%
Inception v3	50	96%	99%	98%	100%	97%	98%
ResNet50	50	94%	100%	97%	100%	99%	98%
VGGNET16	50	90%	98%	95%	98%	96%	95%
VGGNET19	50	91%	98%	96%	98%	95%	96%
SVM (64x64)	NA	84%	90%	95%	92%	88%	90%
Proposed Method	50	96%	100%	98%	100%	100%	99%

Class wise F1-score in comparison with state-of-the-art models shows a robust performance.

**Table 7:** Model performance evaluation and comparison-table with the state-of-the-art models

Method Name	Epoch	Accuracy%	Error%	Precision%	Recall%	F1-Score%
Minhas et al. [1]	1000	94.34	5.66	94.50	94.34	94.14
Inception v3	50	98.41	1.59	98.50	98.41	98.38
ResNet50	50	98.99	1.01	99.00	98.99	98.99
VGGNET16	50	97.46	2.54	97.52	97.46	97.46
VGGNET19	50	98.09	1.91	98.20	98.09	98.07
SVM	NA	86.71	13.29	87.28	86.71	86.80
Proposed method	50	99.26	0.74	99.27	99.26	99.26

The method proposed in Minhas et al. [1] is implemented and evaluated using the same dataset as of the proposed model. Results show a performance gain over the existing state-of-the-art methods. Furthermore, the proposed method shows a performance superiority over competitive models.
